# Improving cognitive mapping by training for people with a poor sense of direction

**DOI:** 10.1186/s41235-020-00238-1

**Published:** 2020-08-17

**Authors:** Toru Ishikawa, Yiren Zhou

**Affiliations:** 1 University of Tokyo, Tokyo, Japan; 2 INIAD Toyo University, 1-7-11 Akabanedai, Kita-ku, Tokyo, 115-0053 Japan

**Keywords:** Spatial cognition, Spatial orientation, Training, Feedback, Individual differences

## Abstract

The skill of spatial learning and orientation is fundamental in humans and differs widely among individuals. Despite its importance, however, the malleability of this skill through practice has scarcely been studied empirically, in contrast to psychometric spatial ability. Thus, this article examines the possibility of improving the accuracy of configurational understanding of the environment by training. A total of 40 adults with a poor sense of direction participated in the experiment; and were randomly assigned to either a condition in which they received feedback only or a condition in which they additionally practiced allocentric spatial updating. Participants walked one route in each session, once a week for 6 weeks, and conducted spatial tasks designed to assess their knowledge of the route. A total of 20 people with an average sense of direction also participated as a comparison group. Results showed that training in allocentric spatial updating improved the accuracy of direction estimates, although the size of the effect was limited: the improvement was not large enough to equate the performance in the groups with a poor versus average sense of direction. The two groups, however, did not differ in spatial skill in mental rotation or path integration. Feedback was effective for improving accuracy in straight-line distance estimates and sketch maps: repeated trials with feedback led to improved accuracy by the sixth session to a level comparable to the group with an average sense of direction. The results show that flexible translation between viewer-centered and environment-centered representations is difficult and not readily trainable, and provide insights into the nature of individual differences in large-scale environmental cognition.

## Significance statement

One of the issues that has attracted attention recently in cognitive research and educational practice is spatial thinking. Spatial ability and skills are shown to play important roles in science, technology, engineering, and mathematics learning (STEM) and everyday activities. In particular, space and spatiality form a fundamental part of our daily cognition and behavior, because we live in, and are surrounded by, space. Spatial orientation in the environment with an accurate understanding of spatial relations and layouts, however, poses difficulty for people with a poor sense of direction. The accuracy and developmental pattern of spatial knowledge acquisition (or cognitive mapping) thus show large individual differences, which offers practical implications for navigation assistance, environmental design, and personnel selection. Despite such large individual differences and significant practical implications, the malleability of the skill of cognitive mapping has scarcely been studied empirically, in stark contrast to the body of literature on the training in spatial ability. This article, therefore, examines how and whether cognitive mapping can be improved by training. Results from the present empirical study provide theoretical insights into the structure and process of spatial knowledge and stimulate discussions on the development of user-adapted navigation systems and spatial awareness.

## Background

### Cognitive mapping and spatial orientation

For humans, who live in and are surrounded by space, it is fundamental to learn about the surrounding environment and stay oriented in it. You daily encounter situations in which you need to know where a destination is, which way to turn at an intersection, or how to make a detour when your usual route is blocked. Adaptive and flexible behavior in the environment depends on an accurate understanding of where you are and which direction you are facing in relation to other objects (physical or conceptual) in space. When you have navigational assistance available such as a map, you still need to consult your knowledge, so that you align the map in the hand with a “map in the head.” Thus, having an accurate mental representation of the environment and being able to manipulate it flexibly are key to efficient spatial reasoning and behavior. Such a psychological process of acquiring, coding, storing, recalling, and decoding information about everyday spatial environments is called *cognitive mapping* (Downs & Stea, 1973). This article looks at the skill of cognitive mapping from the perspective of individual differences, and examines the possibility of improving it by training in people with difficulty in survey learning and spatial orientation in the environment.

A large-scale space, or space at the environmental scale (beyond the vista scale) has unique characteristics: the environment is larger than and surrounds the human body, and cannot be observed in its entirety from a single viewpoint (Ittelson, 1973; Jacobs & Menzel, 2014; Montello, 1993; Wolbers & Wiener, 2014). The difficulty in cognitive mapping, therefore, is comprehensive learning of the environment in the *configurational* and *global* sense, because it requires moving around and mentally integrating views at individual locations into a “network” of spatial relations (Chrastil & Warren, 2014).

Interrelating separately learned places in a common frame of reference constitutes an important step in the acquisition of spatial knowledge in a new environment (Montello, 1998; Siegel & White, 1975), as it signifies a transition from the knowledge of discrete objects and sequences of landmarks (landmark and route knowledge) to the knowledge of spatial relations (survey or configurational knowledge). This step of survey learning poses difficulty, especially for people with a poor sense of direction (Hegarty, Montello, Richardson, Ishikawa, & Lovelace, 2006; Kozlowski & Bryant, 1977), and because of the difficulty, the outcome of cognitive mapping (or “cognitive maps”) differs widely among individuals in terms of metric and configurational accuracy (Ishikawa & Montello, 2006). Past research has explored this topic with respect to the integration of separate routes, for example, two routes sharing a common segment, connected by a third route, or laid out on different levels (Golledge, Ruggles, Pellegrino, & Gale, 1993; Ishikawa & Montello, 2006; Moar & Carleton, 1982; Montello & Pick, 1993).

The difficulty of survey learning has also been discussed in the context of spatial orientation in local and global reference frames. People are good at orienting themselves with respect to local, rather than global, landmarks: for example, they respond more accurately and faster to views aligned with the entrance to a room or parallel to a hallway in a building (Han & Becker, 2014; Marchette, Vass, Ryan, & Epstein, 2014; Meilinger, Riecke, & Bülthoff, 2014; cf. Shine, Valdés-Herrera, Hegarty, & Wolbers, 2016). Spatial orientation in a global framework, in contrast, requires the identification of one’s location and heading with respect to a fixed frame of reference. That is, in addition to distances and directions to other objects in the environment, spatial relations between the referenced objects need to be comprehended. Stated differently, object-to-object spatial relations and individual self-to-object relations should be accessible in a common framework as an allocentric, beyond egocentric, representation.

A major input for such spatial updating is information from self-motion in the environment (vestibular, kinesthetic, motor efference copy, and optic flow information), and in the context of spatial orientation, path integration (or dead reckoning) has been identified to play important roles in insects, birds, and mammals (Etienne & Jeffery, 2004; Gallistel, 1990; Klatzky et al., 1990; Loomis et al., 1993; Mittelstaedt & Mittelstaedt, 1980, 2001). For example, Wolbers, Hegarty, Büchel, and Loomis (2008) discuss human use of self-motion cues to update egocentric internal representations. But because path integration is prone to the accumulation of errors, people fix their positions in reference to landmarks. Zhou and Warren (2015) showed that people rely on landmarks and use path integration only when they find landmark information unreliable. These studies point to the difference in the roles played by self-referenced dead reckoning and externally referenced place learning.

Another important issue to be considered in the discussion of cognitive mapping is individual differences. People differ greatly in the accuracy and developmental pattern of configurational understanding of traveled routes, their performance being correlated with sense of direction (Ishikawa & Montello, 2006), working memory capacity (Weisberg & Newcombe, 2016), and hippocampus volume (Schinazi, Nardi, Newcombe, Shipley, & Epstein, 2013; cf. Weisberg, Newcombe, & Chatterjee, 2019). Importantly for the discussion of the present article, people’s performance, on average, is not found to show much improvement through repetition without any feedback or training (Ishikawa & Montello, 2006).

It poses an important question about malleability: whether and how people can be trained in the skill of cognitive mapping. Can the accuracy of configurational understanding of the environment be improved by training, particularly in people with a poor sense of direction? The issue of trainability has been extensively studied in regard to psychometric spatial ability (e.g., Kail & Park, 1990; Wright, Thompson, Ganis, Newcombe, & Kosslyn, 2008). A meta-analysis conducted by Uttal et al. (2013) shows that training in spatial ability is effective, and that the effect of training is transferrable. However, the issue of trainability has scarcely been studied with respect to cognitive mapping, a major type of spatial thinking that has significance in people’s everyday lives (McKinlay, 2016; National Research Council, 2006; Newcombe, 2010, 2019).

### Training and feedback for cognitive mapping

There are a few examples of empirical studies of the effects of instruction on cognitive mapping, and their results are generally not positive. Wen, Ishikawa, and Sato (2014) examined two methods of instructing people to learn about routes from a video, based on the finding that people with a good sense of direction use a rational combination of verbal, visual, and spatial processing, whereas people with a poor sense of direction mainly rely on verbal processing (Wen, Ishikawa, & Sato, 2013). Survey learning by people with a poor sense of direction was not affected by instruction, either to employ verbal processing (which they spontaneously tend to use) or to employ spatial processing (which people with a good sense of direction use). Moreover, both instructional methods interfered with survey learning in people with a good sense of direction. Likewise, Weisberg and Newcombe (2016) reported that people’s survey learning was not facilitated by the manipulation of their motivation with a monetary incentive for good performance.

This article, recognizing the significance of body-based information in the learning of large-scale environments and comprehension of one’s position and orientation in an environment-centered, not solely body-centered, framework, uses a training method that centers on allocentric spatial updating, namely, practice in updating one’s position and orientation in a pathway completion task and orienting oneself in the allocentric framework of cardinal directions. It is a type of method used in practice in the sport of orienteering (e.g., Kjellström, 1994; Seidman & Cleveland, 2001). Empirical reports suggest a possible link between attention to allocentric spatial relations and performance on spatial orientation, in terms of an increase in the accuracy of direction estimates (Hao, Huang, Song, Kong, & Liu, 2017) and a decrease in wayfinding errors (Hund & Nazarczuk, 2009) through the experience of using cardinal directions. Similarly, people who speak *absolute* languages are good at allocentrically referenced spatial updating (Levinson, 2003, p. 225) because of their constant tracking of bearings in an environment-centered reference system.

In addition to training, this study also looks at feedback, or knowledge of results, which is known to facilitate performance in various domains (Kim & Hamner, 1976; Wagman, McBride, & Trefzger, 2008; Withagen & Michaels, 2005). In the context of cognitive mapping, estimates of distances that are laid out and perceived over the ground (Gibson & Bergman, 1954; Gibson, Bergman, & Purdy, 1955) or traversed in the environment (Allen & Rashotte, 2006) are improved by practice with feedback of correct distances. The researchers suggested that the provision of correct distances helped participants adjust their responses, and not necessarily altered their percepts; nonetheless, these positive effects of feedback on distance perception contrast with the lack of improvement in the accuracy of distance and direction estimates with mere repetition, without feedback or training, observed by Ishikawa and Montello (2006).

Thus, this study examines the effects of training and feedback on the accuracy of spatial knowledge acquired by people with a poor sense of direction. It looks at whether people who practice allocentric spatial updating learn traveled routes more accurately in the metric and configurational sense than people who receive only feedback on their performance. To examine the size of effects, their performances are compared with the performance by people with an average level of sense of direction, who receive no training or feedback. Also, the effects of training and feedback on cognitive mapping are examined in comparison to the developmental pattern of spatial ability (performance on mental rotation) over repeated trials.

### Spatial reasoning and spatial representations

For successful spatial orientation, one needs to flexibly manipulate internal representations, so that one can comprehend spatial relations between objects from any imagined perspective. Research has shown, however, that mental representations of spatial environments have a preferred orientation, such that spatial inference from memory is faster and more accurate when conducted along a specific direction than others; for example, a perspective from which the layout was viewed, a heading direction aligned with the first leg of a traveled path or a grid-like street pattern in the neighborhood, and a viewing direction parallel to an intrinsically salient axis in the array (McNamara & Valiquette, 2004; Mou, McNamara, Valiquette, & Rump, 2004; Werner & Schmidt, 1999). In particular, estimating relative directions between places from memory, by imagining a heading different from the current one (imagined rotation), is difficult to accomplish without actual body rotation (Klatzky, Loomis, Beall, Chance, & Golledge, 1998). By contrast, imagining moving to a new position without a change in heading (imagined translation) is relatively easy (Rieser, 1989). As Klatzky (1998) put it, deriving *ego-oriented* heading in an allocentric representation is easily achieved by imagined translation, whereas deriving *egocentric* heading cannot be achieved accurately by imagined rotation.

These results point to a psychological difference between imagined angular rotation and linear translation, and thus suggest a difference in the processing of direction information and distance information. Past research shows that the accuracy of allocentric direction estimates decreases with the disparity of a respondent’s current heading from a direction to be estimated, and that the accuracy was significantly correlated with the respondent’s sense of direction (Burte & Hegarty, 2012; Sholl, Kenny, & DellaPorta, 2006). Estimates of straight-line distances, on the other hand, do not receive evidence of mental tracing, lacking a significant relationship with decision latency (Burte & Hegarty, 2012). In some studies, the accuracy of straight-line distance estimates does not correlate significantly with the respondent’s sense of direction (Burte & Montello, 2017).

Differences in the knowledge of distance and direction were also observed by Thorndyke and Hayes-Roth (1982), who compared spatial knowledge acquired from maps and direct navigation. People who studied a map of a building (they had never been to the building) estimated straight-line distances between places in the building as accurately as did people who had 6 months’ (or longer) experience of working in the building. However, map learners estimated relative directions less accurately than did workers. Furthermore, both map learners and workers estimated relative directions less accurately in an imagined condition than in an in-situ condition.

### Research questions and hypotheses

This research addresses three questions specifically. First, it examines the extent to which practice in allocentric spatial updating affects spatial orientation and configurational learning in a large-scale environment. Findings from past research on the distinction of imagined rotation and translation (Presson, 1982; Rieser, 1989) and direction and distance estimates (Burte & Hegarty, 2012; Burte & Montello, 2017; Thorndyke & Hayes-Roth, 1982) suggest a possible difference in the sensitivity of direction and distance judgments to training. To the extent that judgments of relative directions involve mental updating of *egocentric* heading by imagined rotation and judgments of straight-line distances do not (can be made in a fixed, imagined *ego-oriented* heading), the training in allocentric spatial updating would improve direction estimates, but not straight-line distance estimates. The size of the improvement, if any, is an empirical question. Findings of positive effects of feedback on the estimates of distances that were directly perceived or traversed in past studies (Allen & Rashotte, 2006; Gibson et al., 1955; Gibson & Bergman, 1954) suggest a possible improvement in the accuracy of route distance estimates through feedback; its effect on the estimates of straight-line distances and directions remains to be examined.

A second question relates to the effect of scale on the perception and cognition of space. Distance and direction knowledge from path integration in a pathway completion training task concerns a relatively small scale (vista space); whereas distance and direction knowledge for places on environmental test routes concerns a larger scale (environmental space). The question of whether training in vista space exerts influence on spatial learning in environmental space is now of interest. Thus, the size of errors of direction judgments in the pathway completion training task is compared with that of direction judgments on environmental test routes.

A third question concerns a comparison of spatial ability assessed by a psychometric spatial test and spatial learning assessed by performance on spatial tasks for environmental routes. This study thus compares the developmental patterns of mental rotation performance with practice (Uttal et al., 2013; Wright et al., 2008) and configurational learning with training and feedback.

## Method

### Participants

A group of 40 adults (19 men and 21 women) with a poor sense of direction participated in the experiment in return for monetary compensation.^1^ The experiment consisted of six sessions, conducted once a week for 6 weeks. Participants’ ages ranged from 19 to 36 years, with a mean (*M*) of 24.9 and a standard deviation (*SD*) of 3.2. They were assigned randomly to either a feedback-only condition (7 men and 13 women) or a training condition (12 men and 8 women). Participants in the feedback-only condition received feedback about their performance on experimental spatial tasks; participants in the training condition engaged in training in allocentric spatial updating.

Another group of 20 adults (19 men and 1 woman) participated as a comparison group, who had an average sense of direction (called the average group hereafter). Their ages ranged from 21 to 25 years (*M* = 23.7, *SD* = 1.0). Details of the experimental conditions and grouping based on sense-of-direction scores are explained below. The research was approved by the departmental ethical review board and informed consent was obtained from the participants.

### Materials

#### Study area

**Fig. 1:**
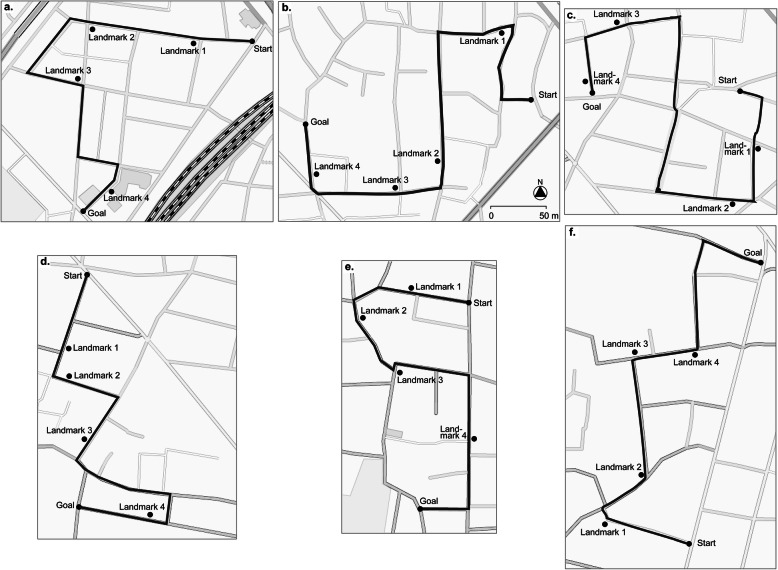
Maps of the study routes. The routes were 450 m in length and contained five turns each. On each route, four landmarks were chosen that were easily recognizable and memorable. The six routes were assigned to the 6 sessions randomly (routes **a**–**f** to sessions 1–6). Participants walked one route in each session and conducted spatial tasks designed to assess their knowledge of the route. The scale bar and north arrow apply to all panels. © OpenStreetMap contributors. http://www.openstreetmap.org/

Six routes in a residential district in the eastern Tokyo area were chosen as a setting for the experiment (Fig. 1). The routes were 450 m in length and contained five turns. On each route, four landmarks were pointed out to participants and these were easily recognizable and memorable, such as buildings, stores, and vending machines. The six routes were assigned to the six experimental sessions randomly: participants walked one route in each session and conducted spatial tasks designed to assess their knowledge about that route. The study area was free of distant views that could be used as navigation clues, and the participants had no prior experience of walking the routes before the experiment.

#### Sense-of-direction scale

Participants’ sense of direction was assessed using the Santa Barbara Sense-of-Direction (SBSOD) scale, which consists of fifteen 7-point Likert-type questions about navigational abilities or preferences (Hegarty, Richardson, Montello, Lovelace, & Subbiah, 2002). It is scored so that a higher score indicates a better sense of direction, ranging in value from 1 to 7. People scoring higher on this scale have been shown to perform better on “survey” tasks, which require an accurate understanding of the environment in terms of metric configurations and layout.

Montello and Xiao (2011) examined scores on the SBSOD scale cross-culturally among 550 respondents in four different countries, and reported a mean score of 4.2 and a standard deviation of 1.1. In the present experiment, the 40 participants with a poor sense of direction were recruited on the condition that their SBSOD scores were below 4; their scores ranged from 1.5 to 3.8 with a mean of 2.9 (*SD* = 0.7), which is more than one *SD* below the cross-cultural mean shown above. The 20 participants in the average group had a mean SBSOD score of 4.5 (*SD* = 1.2), a typical score observed cross-culturally. These values attest to the validity of referring to the two groups as people with a poor and an average sense of direction, respectively.

#### Spatial ability test

Participants took the Card Rotations Test (Ekstrom, French, Harman, & Dermen, 1976), which assesses the ability to rotate mental images. It constitutes one of the major factors of spatial abilities called spatial visualization (McGee, 1979) and relates to map use and spatial orientation in the environment (Hegarty et al., 2006; Liben & Downs, 1993). In the test, participants viewed 20 items, each consisting of 1 criterion card and 8 alternatives, and answered whether the alternative cards are the same as the criterion (rotated into different orientations). They were given 6 min to complete the test, and received 1 point for each correctly identified card and lost 1 point for each wrongly identified card.

#### Training

**Fig. 2:**
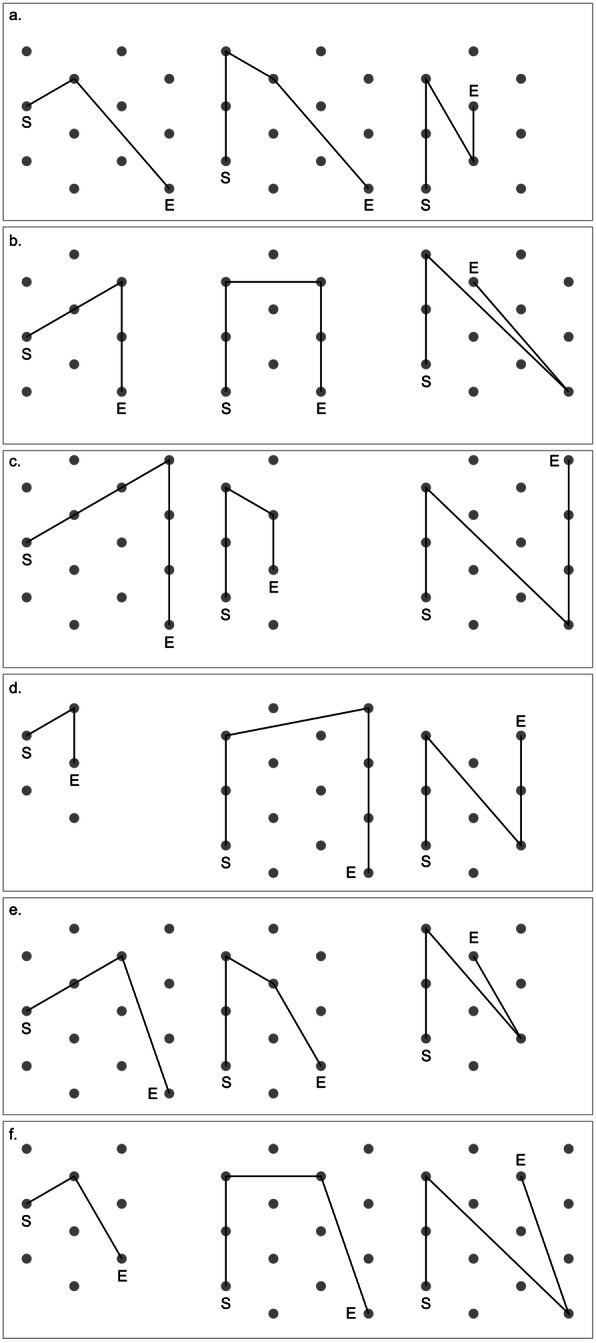
Paths for the training tasks. Participants in the training condition practiced allocentric spatial updating on three paths in each session (paths **a**–**f** assigned to sessions 1–6). Triangle paths are shown in the left column, quadrilateral paths in the middle column, and crossing paths in the right column. Participants walked the paths from the start to the end, blindfolded and guided by the experimenter, and were asked to go directly back to the starting point by themselves, still blindfolded. When they indicated that they reached the starting point, they were asked to point in the direction of north. The gray dots, used for scaling, are placed 1.5 m apart. S = start; E = end

Participants in the training condition practiced spatial updating in a pathway completion task and allocentric spatial orientation with respect to cardinal directions. As in the study of Klatzky et al. (1990), we constructed a total of 18 training paths by varying the number of segments and turns (Fig. 2). A first group of paths were configured so that they formed a triangle when participants went back to the starting point. A second group of paths formed a quadrilateral. A third group of paths were configured so that the final segment that led to the starting point crossed a previous segment. Three paths were selected randomly (one triangle, one quadrilateral, and one crossing) and assigned to each of the six sessions for training.

Participants walked, wearing a blindfold and being guided by the experimenter, a training path, and were asked to go directly back to the starting point by themselves, still blindfolded. Participants stopped and told the experimenter when they thought they reached the starting point. For each path, participants also orientated themselves with respect to cardinal directions. At the start of each path, the direction of north was pointed out to participants. When they walked the path and indicated that they reached the starting point, they were asked to point in the direction of north. The experimenter recorded the locations where participants stopped and the directions that they pointed to.

After completing the pathway completion and north-pointing tasks, participants removed the blindfold and saw how well they had performed, by viewing and comparing the estimated and correct locations of the starting point, and the estimated and correct directions of north, respectively.^2^ They repeated the training tasks twice on the same path. When participants completed the two sets (or trials) of practices for a triangle path, they conducted two sets of practices for a quadrilateral path, and then two sets of practices for a crossing path. Thus, in each session, participants in the training condition practiced two sets of allocentric spatial updating on each of three paths.

#### Measured variables of spatial learning

Participants’ learning of test routes was assessed in terms of the accuracy of their direction and distance estimates and sketch maps.

##### Direction estimates

Participants estimated relative directions between four landmarks on each route. To do that, participants imagined being at one landmark as they had encountered it while walking the route, and indicated the direction to another landmark. Participants were given sheets of A4-sized paper, on which a circle with a radius of 2 cm was drawn; the center of the circle represented the imagined location, and an upward line drawn from the center represented the facing direction at that landmark. They estimated the direction from the landmark to another landmark by drawing a line from the center to the circumference of the circle. We examined the mean absolute error of six direction estimates for each participant.

##### Distance estimates

Participants estimated distances between four landmarks on each route in terms of route distances and straight-line distances, in comparison to a standard distance (which they had walked at the beginning of each experimental session). On a sheet of A4-sized paper, a line that represented the length of the standard distance was printed; on each of the lines printed below the standard line, participants placed a mark to indicate the ratio of a test distance to the standard distance. The accuracy of distance estimates was assessed in terms of correlation between estimated and correct distances and the ratio of an estimated distance to a correct distance.

##### Sketch maps

Participants drew a sketch map of each route traveled on a blank sheet of A4-sized paper, depicting the shape of the route and the locations of the start, goal, four landmarks, and five turns, as accurately as possible. The accuracy of sketch maps was assessed in terms of bidimensional correlation, which measures the degree of correspondence between the coordinates of anchor points (in the present case, the start, goal, four landmarks, and five turns) on a sketch map and a correct map (Tobler, 1994). Values of bidimensional correlation coefficients range from 0 to 1, with a larger value indicating a greater degree of correspondence.

#### Feedback on experimental tasks

After completing the spatial tasks for each test route, participants received feedback on their performance.^3^ They were shown the correct answers of direction and distance estimates and a cartographic map of the route, together with their own estimates and sketch maps. They talked about their responses with the experimenter; for example, what they found difficult in the learning of the route, what they thought about their performances, and why their responses differed from the correct directions, distances, and map.

### Procedure

The experiment was conducted once a week for 6 weeks consecutively (six sessions in total). At the beginning of each session, participants viewed and walked a standard distance, a straight path with a length of 32 m. Then, participants in the training condition engaged in the training phase by practicing allocentric spatial updating, in a park located near the study routes (participants in the feedback-only condition did not receive training).

Participants were then taken to the starting point of the first route, and started walking the route guided by the experimenter. At the goal location, participants estimated directions, route distances, and straight-line distances between landmarks on the route, and drew a sketch map of the route. After completing these spatial tasks for the route, participants received feedback on their performance. At the end of the session, participants took the Card Rotations test. The session took 40 min on average for the feedback-only condition and 60 min for the training condition.

The same procedure was repeated for the other five sessions. The assignment of study routes and training paths to the six sessions was randomly determined, and fixed across participants. Participants received no information about the spatial relations between the study routes (i.e., how the six routes were located in relation to each other).

Participants in the comparison group, the average group, traveled the first route that the participants with a poor sense of direction traveled, and estimated directions, route distances, and straight-line distances and drew a sketch map for the route with the same procedure as above.

## Results

We first look at performances by participants in the two experimental conditions (with a poor sense of direction) to examine the effects of feedback and training. We then examine their performances in more detail in comparison to participants with an average sense of direction (toward the end of this section).

### Comparability of participants

To ensure the equivalence of participants in the two experimental conditions, we compared their scores on the SBSOD scale and the Card Rotations Test in the first session (an alpha level of .05 was used in all analyses below). There was not a significant difference between the feedback-only and training conditions in the mean scores of SBSOD, *t* (38) = 0.38, *p* = .705, *d* = 0.12, or Card Rotations, *t* (38) = 1.01, *p* = .320, *d* = 0.32.

### Performance on training tasks

Participants’ performance on the training tasks in each session was assessed in terms of distance and direction deviations in pathway completion and direction errors in north-pointing; specifically, a mean deviation in distance between estimated and correct start locations and a mean angular deviation of estimated and correct return paths, and a mean absolute angular error of estimates of the direction of north. These measures were examined in a repeated measures analysis of variance (ANOVA) with trial (first and second sets of practices in each session) and session number (sessions 1–6) as within-subject variables.

**Fig. 3:**
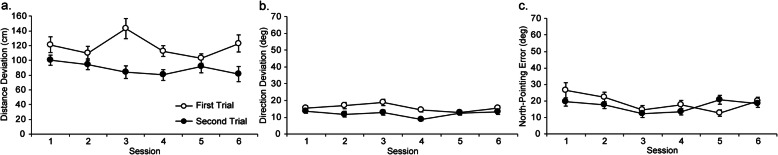
Results of a repeated measures analysis of variance for the performance on training tasks: distance and direction deviations in pathway completion (**a** and **b**, respectively) and direction errors in north pointing (**c**). Vertical lines depict standard errors of the means

A repeated measures ANOVA on distance deviations revealed a significant main effect of trial, *F* (1, 19) = 32.52, *p* < .001, η_p_^2^ = 0.63, and a significant interaction between trial and session number, *F* (5, 95) = 2.83, *p* = .020, η_p_^2^ = 0.13 (Fig. 3a). In post hoc *t* tests (Bonferroni), the distance deviation was smaller in the second trial of practice than the first trial in sessions 3, 4, and 6. This indicates that distance judgments in the second trial in each session with feedback were more accurate than those in the first trial, which were less stable and fluctuated between sessions. The main effect of session number was nonsignificant, *F* (5, 95) = 0.97, *p* = .438, η_p_^2^ = 0.05.

A repeated measures ANOVA on direction deviations revealed significant main effects of trial, *F* (1, 19) = 13.51, *p* = .002, η_p_^2^ = 0.42, and session number, *F* (5, 95) = 2.46, *p* = .039, η_p_^2^ = 0.11 (Fig. 3b). The interaction between the two variables was nonsignificant, *F* (5, 95) = 1.53, *p* = .187, η_p_^2^ = 0.07. With respect to the effect of trial, direction deviations were smaller on average in the second trial of practice than in the first trial (*M*s = 12.05° and 15.65°, respectively). A trend analysis for session number did not reveal a significant linear, *F* (1, 19) = 2.17, *p* = .157, η_p_^2^ = 0.10; quadratic, *F* (1, 19) = 0.58, *p* = .454, η_p_^2^ = 0.03; or cubic, *F* (1, 19) = 3.44, *p* = .079, η_p_^2^ = 0.15, trend.

A repeated measures ANOVA on north-pointing errors revealed a significant main effect of session number, *F* (5, 95) = 3.08, *p* = .013, η_p_^2^ = 0.14, and a significant interaction between trial and session number, *F* (5, 95) = 2.70, *p* = .025, η_p_^2^ = 0.12 (Fig. 3c). The main effect of trial was nonsignificant, *F* (1, 19) = 2.98, *p* = .101, η_p_^2^ = 0.14. A trend analysis for session number revealed a significant quadratic trend, *F* (1, 19) = 7.96, *p* = .011, η_p_^2^ = 0.30, but the trend did not point to an improvement over the six sessions; the direction errors in the first and sixth sessions did not differ significantly in either the first trial, *t* (19) = 0.05, *p* = .959, *d*_*z*_ = 0.01, or the second trial, *t* (19) = 0.20, *p* = .846, *d*_*z*_ = 0.04.

### Performance on spatial tasks on test routes

#### Direction estimates

**Fig. 4:**
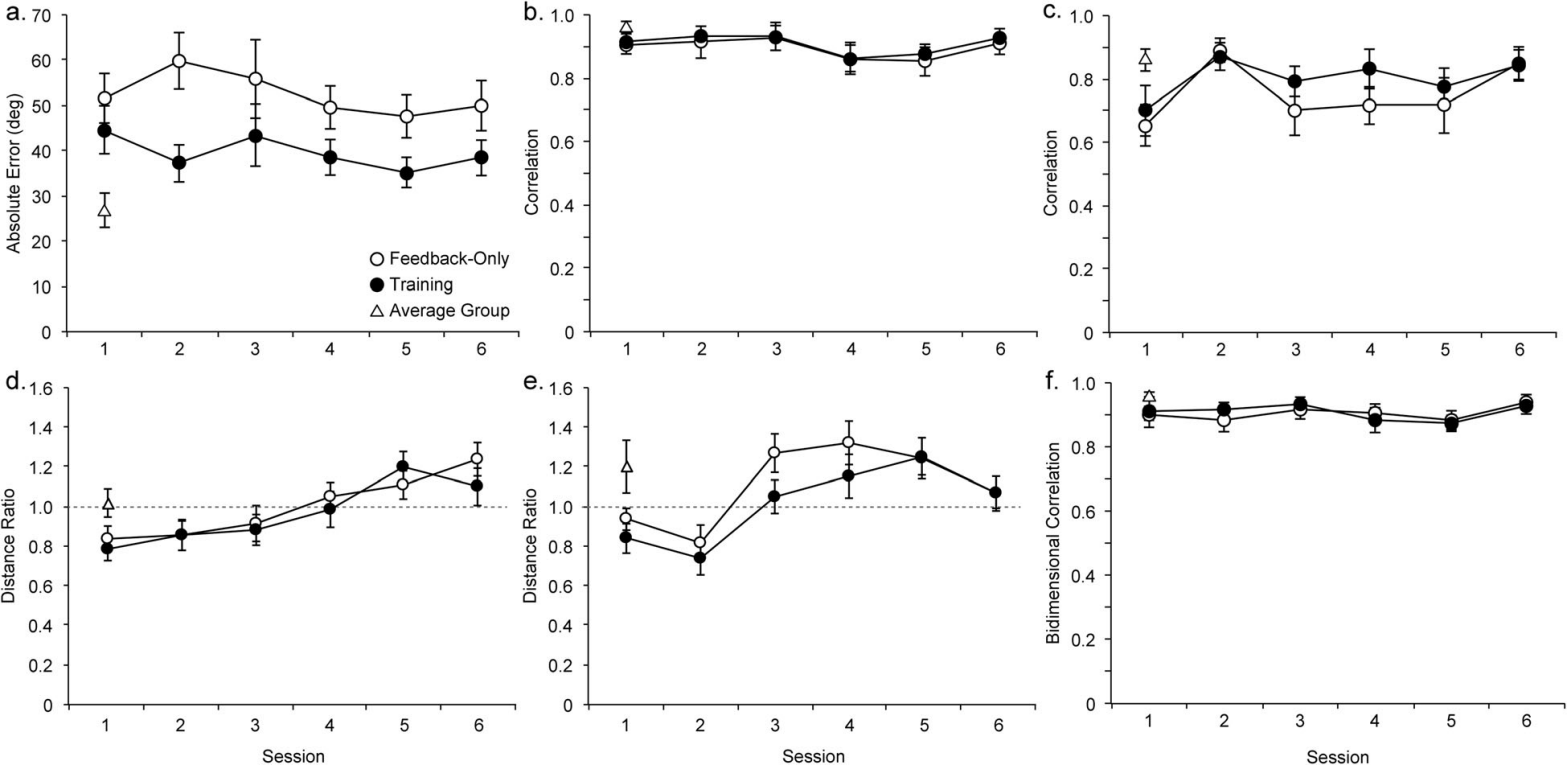
Results of a mixed analysis of variance for direction estimates (**a**), distance correlations for route distances (**b**) and straight-line distances (**c**), distance ratios for route distances (**d**) and straight-line distances (**e**), and sketch maps (**f**). Correlations for distance estimates and sketch maps are shown in terms of *r*. Values transformed into Fisher’s z were analyzed. The average group estimated directions and distances and drew sketch maps for the first route (their performance is shown by triangles). Vertical lines depict standard errors of the means

Absolute errors of participants’ direction estimates were examined in a mixed ANOVA with condition (feedback-only and training) as a between-subject variable and session number (sessions 1–6) as a within-subject variable. There was a significant main effect of condition, *F* (1, 38) = 6.88, *p* = .013, η_p_^2^ = 0.15, showing that participants in the training condition estimated directions more accurately than participants in the feedback-only condition (*M*s = 39.57° and 52.32°, respectively; Fig. 4a). The effect size (the standardized mean difference, Cohen’s *d*, in absolute errors between the feedback-only and training conditions) was *d* = 0.83.^4^ The main effect of session number, *F* (5, 190) = 1.00, *p* = .419, η_p_^2^ = 0.03, and interaction between condition and session number, *F* (5, 190) = 0.65, *p* = .665, η_p_^2^ = 0.02, were nonsignificant.

Although the main effect of session number was not significant, a trend analysis revealed a significant linear trend, *F* (1, 38) = 4.40, *p* = .043, η_p_^2^ = 0.10, indicating an improvement over sessions. The standardized mean difference in absolute errors between the first and last sessions was *d*_*z*_ = − 0.07 for the feedback-only condition (a decrease in errors from 51.60° to 49.84°), and *d*_*z*_ = − 0.25 for the training condition (from 44.57° to 38.60°).

Direction errors on the test routes among participants in the training condition were compared with direction deviations in the pathway completion training task, to examine direction estimates at two different spatial scales, yielding a significant difference, *F* (3, 57) = 57.50, *p* < .001, η_p_^2^ = 0.75. Post hoc *t* tests (Bonferroni) showed that the mean error on test routes (39.57°) was larger than errors for pathway completion (15.65° and 12.05° for the first and second trials, respectively) and north-pointing (17.95°), *t* (19) = 7.62, 9.36, and 6.72, respectively, *p* < .001; *d*_*z*_ = 1.70, 2.09, and 1.50. This particularly shows that direction learning on a test route at the environmental scale was more difficult than that on a training path at vista scale.

#### Distance estimates: correlation

For participants’ route distance and straight-line distance estimates, correlation between estimated and correct distances (with Fisher’s *r*-to-*z* transformation applied) was examined in a mixed ANOVA with condition as a between-subject variable and session number as a within-subject variable.

##### Route distances

There was a significant main effect of session number, *F* (5, 190) = 3.17, *p* = .009, η_p_^2^ = 0.08. The main effect of condition, *F* (1, 38) = 0.37, *p* = .548, η_p_^2^ = 0.01, and interaction between condition and session number, *F* (5, 190) = 0.05, *p* = .999, η_p_^2^ = 0.00, were nonsignificant.

A trend analysis for session number revealed a significant cubic trend, *F* (1, 38) = 10.18, *p* = .003, η_p_^2^ = 0.21. The significant trend did not, however, point to an improvement over the six sessions (Fig. 4b); the distance correlations for the first and last sessions did not differ significantly, *t* (39) = 0.47, *p* = .642, *d*_*z*_ = 0.07. The performance was good overall across the sessions, with a mean distance correlation of .91.

##### Straight-line distances

There was a significant main effect of session number, *F* (5, 190) = 5.44, *p* < .001, η_p_^2^ = 0.13. The main effect of condition, *F* (1, 38) = 0.88, *p* = .353, η_p_^2^ = 0.02, and interaction between condition and session number, *F* (5, 190) = 0.63, *p* = .676, η_p_^2^ = 0.02, were nonsignificant.

A trend analysis for session number revealed a significant cubic trend, *F* (1, 38) = 14.92, *p* < .001, η_p_^2^ = 0.28, pointing to an improvement over the six sessions. The distance correlations for the first and last sessions (*M*s = .68 and .85, respectively) differed significantly, *t* (39) = 3.23, *p* = .003, with a medium effect size of *d*_*z*_ = 0.51 (Fig. 4c).

#### Distance estimates: ratio to correct distance

Distance estimates were also examined in terms of the ratio to a correct distance (an estimated distance divided by a correct distance), in a mixed ANOVA with condition as a between-subject variable and session number as a within-subject variable.

##### Route distances

There was a significant main effect of session number, *F* (5, 190) = 18.30, *p* < .001, η_p_^2^ = 0.32. The main effect of condition, *F* (1, 38) = 0.20, *p* = .657, η_p_^2^ = 0.01, and interaction between condition and session number, *F* (5, 190) = 0.88, *p* = .493, η_p_^2^ = 0.02, were nonsignificant.

A trend analysis for session number revealed a significant cubic trend, *F* (1, 38) = 4.92, *p* = .033, η_p_^2^ = 0.11, pointing to an improvement over the six sessions (Fig. 4d). Post hoc *t* tests (Bonferroni) showed that the ratio was significantly different from 1 in the first three sessions, *t* (39) < − 4.80, *p* < .001, *d*_*z*_ > 0.76, but not in the last three sessions, *t* (39) > − 2.27, *p* > .029, *d*_*z*_ < 0.36.

##### Straight-line distances

There was a significant main effect of session number, *F* (5, 190) = 17.97, *p* < .001, η_p_^2^ = 0.32. The main effect of condition, *F* (1, 38) = 0.66, *p* = .599, η_p_^2^ = 0.02, and interaction between condition and session number, *F* (5, 190) = 0.72, *p* = .611, η_p_^2^ = 0.02, were nonsignificant.

A trend analysis for session number revealed a significant cubic trend, *F* (1, 38) = 21.83, *p* < .001, η_p_^2^ = 0.36, and the trend pointed to an improvement over the six sessions (Fig. 4e). In post hoc *t* tests (Bonferroni), the ratio was significantly different from 1 in the first two sessions, *t* (39) < − 2.82, *p* < .007, *d*_*z*_ > 0.45, but not in the last four sessions, *t* (39) < 2.76, *p* > .009, *d*_*z*_ < 0.44.

#### Sketch maps

Participants’ sketch maps were analyzed through bidimensional regression and Monte Carlo simulation.

##### Bidimensional regression

Bidimensional correlation coefficients for participants’ sketch maps (with Fisher’s *r*-to-*z* transformation applied) were examined in a mixed ANOVA with condition as a between-subject variable and session number as a within-subject variable. There was a significant main effect of session number, *F* (5, 190) = 3.09, *p* = .010, η_p_^2^ = 0.08. The main effect of condition, *F* (1, 38) = 0.08, *p* = .776, η_p_^2^ = 0.00, and interaction between condition and session number, *F* (5, 190) = 0.64, *p* = .671, η_p_^2^ = 0.02, were nonsignificant.

A trend analysis for session number revealed a significant cubic trend, *F* (1, 38) = 8.59, *p* = .006, η_p_^2^ = 0.18, pointing to an improvement over the six sessions. The bidimensional correlation coefficients for the first and last sessions (*M*s = .91 and .93, respectively) differed marginally significantly, *t* (39) = 1.86, *p* = .070, with a small effect size of *d*_*z*_ = 0.29 (Fig. 4f).

##### Monte Carlo simulation

We simulated participants’ map drawing for a test route using the observed values of their distance and direction estimates on the route. Specifically, starting from the first landmark on the route, the *x* and *y* coordinates of the next landmark can be determined based on the ratio of an estimated straight-line distance to the correct distance and the absolute error of a direction estimate between the two landmarks. We assumed a normal distribution for distance and direction estimates, respectively, with a mean and a standard deviation observed for the participants’ straight-line distance and relative direction estimates, and probabilistically generated the coordinates of the four landmarks on the route through Monte Carlo simulation with 1000 iterations. We then computed a mean bidimensional correlation coefficient over the 1000 iterations, and compared it with the observed performance of our participants. Since the main effect of session number was significant in the mixed ANOVA, we tested a mean bidimensional correlation for the first session and the last session, for the feedback-only and training conditions, respectively.

As shown in Table 1, simulated bidimensional correlation values were significantly smaller than the values obtained from our participants’ sketch maps. That is, the accuracy of our participants’ (with a poor sense of direction) sketch maps was greater than was simulated on the basis of their straight-line distance and direction estimates. Considering the fact that the accuracy of straight-line distance estimates was high (a ratio not significantly different from 1 in the sixth session), one can regard the poorer performance of simulated map drawing as reflecting higher accuracy of spatial relations depicted on participants’ sketch maps than revealed by their relative direction judgments.

**Table 1:** Mean bidimensional correlations for participants’ sketch maps and simulations

	Participants	Simulation	*t* (19)	*p*
First session				
Feedback-only	.90	.74	3.96	< .001
Training	.91	.74	5.54	< .001
Sixth session				
Feedback-only	.94	.84	4.39	< .001
Training	.93	.84	3.33	.004
Average group	.95	.75	9.64	< .001

#### Card Rotations scores

Participants’ scores on the Card Rotations Test were examined in a mixed ANOVA with condition as a between-subject variable and session number as a within-subject variable. There was a significant main effect of session number, *F* (5, 190) = 55.37, *p* < .001, η_p_^2^ = 0.59. The main effect of condition, *F* (1, 38) = 1.33, *p* = .257, η_p_^2^ = 0.03, and interaction between condition and session number, *F*(5, 190) = 0.16, *p* = .977, η_p_^2^ = 0.00, were nonsignificant.

**Fig. 5:**
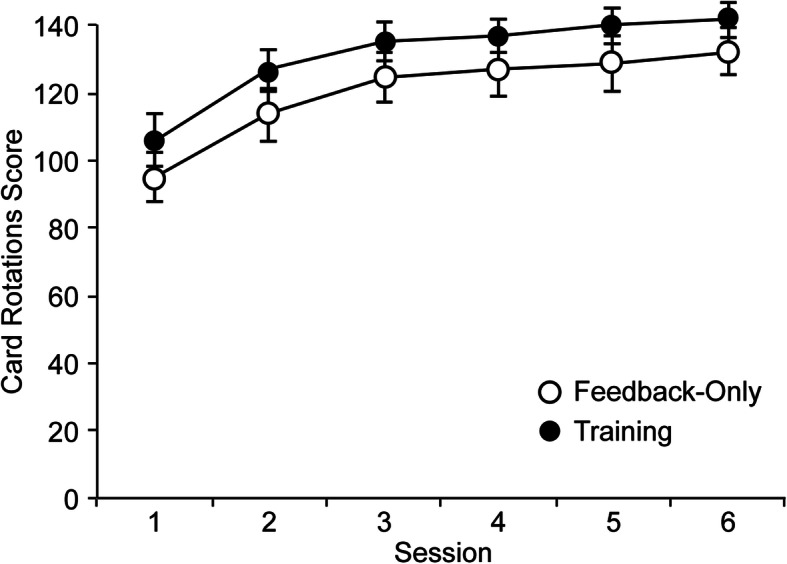
Results of a mixed analysis of variance for Card Rotations scores. Vertical lines depict standard errors of the means

A trend analysis for session number revealed significant linear, quadratic, and cubic trends, *F* (1, 38) = 93.57, *p* < .001, η_p_^2^ = 0.71; *F* (1, 38) = 55.40, *p* < .001, η_p_^2^ = 0.59; and *F* (1, 38) = 9.01, *p* = .005, η_p_^2^ = 0.19, respectively. Mean Card Rotations scores increased significantly from the first to the second sessions, *t* (39) = 7.71, *p* < .001, *d*_*z*_ = 1.22; and from the second to the third sessions, *t* (39) = 4.54, *p* < .001, *d*_*z*_ = 0.72 (Fig. 5).

#### Male-female differences

No significant male-female differences were observed among the present participants with a poor sense of direction, except for route distance estimates in the first session, *t* (38) = 2.18, *p* = .036, *d* = 0.69, with women performing better than men.^5^

#### Correlation between spatial tasks

The relationship among participants’ performances on the spatial tasks and sense of direction is shown in Table 2. The weak correlations with sense of direction show that the participants were recruited from a group of people with a poor sense of direction (their sense-of-direction scores are in a restricted range). The three measures, direction estimates, distance estimates, and sketch maps, correlated partially, especially straight-line distance estimates and sketch maps, but they tap into different aspects of cognitive mapping.

**Table 2:** Correlations among spatial tasks and sense of direction

	1	2	3	4
1. Sense of direction				
2. Direction estimates	−.10			
3. Route distance estimates	−.25	−.12		
4. Straight-line distance estimates	.04	−.30	.24	
5. Sketch maps	.13	−.22	.29	.48**

*Note:* Measures for direction estimates are absolute errors in degrees, for distance the estimates are distance correlations, and for sketch maps they are bidimensional correlation coefficients

**p < .01

### Comparison with the average group

In the preceding section, we compared performances for the feedback-only and training conditions. To examine further the effects of feedback and training on configurational learning for people with a poor sense of direction, we next compared their performances with the performance by the average group (see Fig. 4).

#### Direction estimates

Since the main effect of condition was significant in the mixed ANOVA as reported above, the mean absolute error across the six sessions was computed for each condition separately, the feedback-only condition (*M* = 52.32°, *SD* = 17.66°) and the training condition (*M* = 39.57°, *SD* = 12.70°). Their errors were larger than that for the average group (*M* = 27.06°, *SD* = 17.89°), *t* (38) = 4.49, *p* < .001, *d* = 1.42; and *t* (38) = 2.55, *p* = .015, *d* = 0.80, respectively. This shows that although participants in the training condition performed better than those in the feedback-only condition, participants in the two conditions did not perform as well as participants in the average group. That is, neither training nor feedback improved the accuracy of direction estimates by people with a poor sense of direction to a level comparable to the average group.

### Distance estimates: correlation

#### Route distances

Since no significant main or interaction effects of condition were observed in the mixed ANOVA and the developmental trend did not point to an improvement, a mean distance correlation across the six sessions was computed, with the feedback-only and training conditions collapsed (*M* = .91, *SD* = .36). The mean correlation was smaller than that for participants in the average group (*M* = .96, *SD* = .59), *t* (58) = − 3.53, *p* < .001, *d* = 0.97, showing that neither feedback nor training improved the accuracy of route distance estimates by people with a poor sense of direction to a level comparable to the average group.

#### Straight-line distances

Since the main effect of session number was significant in the mixed ANOVA, a mean distance correlation was computed for the first session and the last session, with the feedback-only and training conditions collapsed. The mean correlation for the first session (*M* = .68, *SD* = .50) was smaller, but that for the last session (*M* = .85, *SD* = .54) was not different, than that for the average group (*M* = .85, *SD* = .39), *t* (58) = − 2.99, *p* < .001, *d* = 0.82; and *t* (58) = 0.00, *p* = .997, *d* = 0.00, respectively. That is, by the sixth session, participants with a poor sense of direction were able to estimate straight-line distances as accurately as participants in the average group did. Namely, repeated trials with feedback improved the accuracy of their straight-line distance estimates to a level comparable to the average group.

### Distance estimates: ratio to correct distance

#### Route distances

Since the main effect of session number was significant in the mixed ANOVA, a mean distance ratio was computed for the first session and the last session, with the feedback-only and training conditions collapsed. As described above, the mean distance ratio differed from 1 in the first session (*M* = 0.78, *SD* = 0.25), but became equivalent to 1 in the sixth session (*M* = 1.11, *SD* = 0.37). For participants in the average group, the mean distance ratio (*M* = 1.02, *SD* = 0.30) was not significantly different from 1, *t* (19) = 0.29, *p* = .776, *d*_*z*_ = 0.06. That is, by the end of the sixth session, repeated trials with feedback improved route distance estimates by people with a poor sense of direction to a level comparable to the average group.

#### Straight-line distances

Since the main effect of session number was significant in the mixed ANOVA, a mean distance correlation was computed for the first session and the last session, with the feedback-only and training conditions collapsed. As described above, the mean distance ratio differed from 1 in the first session (*M* = 0.90, *SD* = 0.35), but became equivalent to 1 in the sixth session (*M* = 1.07, *SD* = 0.37). For participants in the average group, the mean distance ratio (*M* = 1.20, *SD* = 0.56) was not significantly different from 1, *t* (19) = 2.06, *p* = .054, *d*_*z*_ = 0.46. That is, by the end of the sixth session, repeated trials with feedback improved straight-line distance estimates by people with a poor sense of direction to a level comparable to the average group.

### Sketch maps

Since the main effect of session number was significant in the mixed ANOVA, a mean bidimensional correlation was computed for the first session and the last session, with the feedback-only and training conditions collapsed. The mean bidimensional correlation for the first session (*M* = .91, *SD* = .43) was smaller, but that for the last session (*M* = .93, *SD* = .45) was not different, than that for the average group (*M* = .95, *SD* = .35), *t* (58) = − 2.70, *p* = .009, *d* = 0.74; and *t* (58) = − 1.02, *p* = .310, *d* = 0.28, respectively. That is, by the sixth session, participants with a poor sense of direction were able to draw sketch maps as accurately as participants in the average group did. Namely, repeated trials with feedback improved the accuracy of their sketch maps to a level comparable to the average group.

As in the analysis above, we conducted a Monte Carlo simulation of sketch maps for the average group (Table 1). The bidimensional correlation value from the simulation was significantly smaller than the value obtained from their sketch maps, showing that the accuracy of the average group’s sketch maps was greater than was simulated on the basis of their straight-line distance and direction estimates.

## Discussion

This article examined the possibility of improving performance on configurational understanding of the environment by people with a poor sense of direction through training in allocentric spatial updating. Now we discuss the results from two major viewpoints: (1) effects of training and feedback and (2) different patterns of effects for direction estimates and straight-line distance estimates.

First, the present results clarified the relationship between sense of direction and cognitive mapping in regard to configurational learning. Our participants, with a poor sense of direction, had difficulty with survey learning in the environment, specifically, acquiring accurate knowledge of the configurations of traveled routes and judging spatial relations between places on the routes accurately and flexibly. Their performance in the first session on estimating relative directions and straight-line distances and drawing sketch maps was poorer than that of participants with an average sense of direction. Sense of direction thus closely relates to configurational understanding of the environment.

In the discussion of cognitive mapping, configurational understanding (in the strict sense) and metric (or quantitative) understanding of traveled routes should be carefully contrasted. In particular, estimates of distances in a straight line and along a route need to be distinguished, as the latter can be based on a (one-dimensional) understanding of the amount of displacement, not the shape of the route in two dimensions. Our participants with a poor sense of direction estimated route distances correctly throughout the sessions (with a correlation of .91). In terms of the ratio to a correct distance, their route distance estimates became comparable to the average group over sessions (a ratio not significantly different from 1). Thus, repeated trials with feedback increased the accuracy of distance judgments along traveled routes.

The measures of configurational learning, direction estimates, straight-line distance estimates, and sketch maps, were divided into two types based on sensitivity to the experiential intervention of feedback and training. The accuracy of direction estimates was improved by the training in allocentric spatial updating: errors decreased from 52.32° to 39.57° with training, although the improvement was not large enough to equate their performance with that of the average group (an error of 27.06°).

By contrast, for straight-line distances and sketch maps, the provision of feedback only was found to be effective: repeated trials with feedback improved the accuracy by the sixth session to a level comparable to the average group (a distance correlation of .85 and a map correlation of .95). Participants in the training and feedback-only conditions did not differ in their performance, however. Namely, the training in allocentric spatial updating did not have an effect over and above the effect of feedback.

The patterns of development over trials for the measures of cognitive mapping (Fig. 4) are different from that for the psychometric spatial ability of mental rotation. Participants’ scores on the Card Rotations Test improved steadily with repeated practice, yielding a typically observed negatively accelerated learning curve (Fig. 5). The Card Rotations scores were not affected by the experimental conditions, being equivalent in the feedback-only and training conditions. In particular, the rapid and steady improvement in mental rotation performance indicates that the changes in the accuracy of cognitive mapping as observed above, either with training or feedback, therefore, cannot be associated with the changes in the basic spatial skill in mental rotation.

Second, the two measures of configurational understanding of environmental routes, direction estimates, and straight-line distance estimates, show important differences in their responses to training and feedback. Direction estimates were not affected by feedback, with no changes in the accuracy over repeated trials with feedback only. Through the training in allocentric spatial updating, however, the accuracy of direction estimates improved, both in comparison to the feedback-only condition and over the course of repeated trials with training. Thus, the practice of updating one’s position and orientation in an allocentric frame of reference (training tasks) exerts influence on the process of judging relative directions from an imagined perspective (experimental tasks). Nonetheless, the size of the effect of training is limited. The accuracy of direction estimates improved by training to some extent, but not to the level equivalent to participants with an average sense of direction. Moreover, direction errors on large-scale test routes and small-scale training paths were different in size. Mean direction errors are more than twice larger on the environmental test routes (39.57°) than on the training paths (around 15° for pathway completion and north-pointing), suggesting the significance of spatial scale on spatial cognition (Learmonth, Nadel, & Newcombe, 2002; Newcombe, 2019; to be discussed in more detail below).

By contrast, straight-line distance estimates were improved by feedback: through repeated trials with feedback, our participants with a poor sense of direction were able to estimate straight-line distances as accurately as the average group did. The training in allocentric spatial updating, however, did not affect straight-line distance estimates. These findings point to the distinction in the processes of understanding relative directions and straight-line distances, the former involving the derivation of egocentric, as opposed to ego-oriented, bearing in an allocentric representation from imagined rotation (Klatzky, 1998; Klatzky et al., 1998). Being able to judge relative directions flexibly from memory thus characterizes good survey learners. In this sense, the trait is aptly called sense of “direction.”

The accuracy of sketch maps was similarly improved by feedback. An examination of the orientations of the participants’ sketch maps showed that 89% of the maps were aligned with the facing direction at the start (i.e., the first segment of a route was drawn upward on these maps). The remaining maps were aligned with one of the other route segments, mostly the last segment. These results indicate that a mental representation having a preferred orientation is not a problem for distance judgment and sketch mapping, because they can be performed from a fixed, single perspective. The existence of a preferred orientation, however, poses extra difficulty for relative direction judgment, especially for people with a poor sense of direction, because of the necessity of imagining multiple different viewpoints and perspectives. Simulation results also show that spatial relations are depicted more accurately on sketch maps than revealed by relative direction judgments. Importantly, this mental perspective taking in environmental space is different from mental rotation assessed by a spatial test and spatial updating on a vista-scale path.

Findings in the neuroscience literature (although not the focus of this article) also suggest the distinction of the processing of direction and distance information. Head direction cells work as a “compass” for maintaining orientation and heading (Dumont & Taube, 2015, p. 86), and grid cells are modulated by distance information and provide a spatial “metric” for locational updating (Dumont & Taube, 2015, p. 87). In particular, head direction cells are considered to play fundamental roles in spatial tasks that require environment-centric representations (Chadwick, Jolly, Amos, Hassabis, & Spiers, 2015, p. 90) and cognitive map–like strategies (Dumont & Taube, 2015, p. 89). The dissociation of direction and distance information has also been shown in a lesion study. People with vestibular deficits performed worse on the estimation of turn angles during blindfolded walking, but they performed as well as people without deficits on the estimation of linear displacements (Glasauer, Amorim, Viaud-Delmon, & Berthoz, 2002).

## Conclusion

Our results provide further insights into the discussion of people’s spatial knowledge of large-scale environments by extending the line of research from the perspective of individual differences. Past research has qualified the concept of cognitive maps in regard to metric and geometric accuracy of spatial judgments (Chrastil & Warren, 2014; Ishikawa & Montello, 2006; Schinazi et al., 2013; Weisberg & Newcombe, 2016; Weisberg, Schinazi, Newcombe, Shipley, & Epstein, 2014); especially, the existence of large individual variations in the accuracy and developmental patterns runs counter to the notion of spatial knowledge as a unitary, map-like representation (a strong interpretation of the “maps in the head” metaphor). The present findings add to the non-unitary view on spatial knowledge by revealing a psychological difference in the processing of distance and direction information. Judgments of distance and direction by people with a poor sense of direction differ in sensitivity to the intervention of feedback and training.

**Fig. 6:**
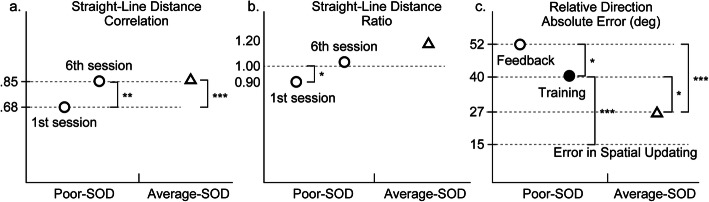
Schematic illustrations of the effects of feedback and training on straight-line distance estimates (distance correlations (**a**) and ratios (**b**)) and relative direction estimates (**c**). SOD, sense of direction. The accuracy of straight-line distance estimates was improved to the level equivalent to the average-SOD group over the course of repeated trials with feedback. Training improved the performance on relative direction judgments to a certain degree, but did not negate the difference between the poor- and average-SOD groups. *p < .05; **p < .01; ***p < .001

Our participants adjusted the accuracy of their estimates of straight-line distances to the level equivalent to people with an average sense of direction, over the course of repeated trials with feedback (Fig. 6a and b). Although judgments of route distances tap into metric understanding of displacement along the line of locomotive movement, and not specifically two-dimensional configurational understanding of the route, their accuracy (the ratio to a correct distance) was also improved by feedback. Notable is the insensitivity of distance judgments to training in allocentric spatial updating, a finding that contrasts with the sensitivity of direction judgments to the training. At the same time, it should be noted that the effect of training is limited—it enhances the performance on relative direction judgments to a certain, significant degree, but does not negate the difference between the two groups of people with a poor and average sense of direction (Fig. 6c).

The two groups, however, did not differ in the spatial skill in mental rotation or path integration. Our participants’ (with a poor sense of direction) mental rotation scores (*M* = 100.35, *SD* = 33.56) are not significantly different from the mean score calculated in the total group of participants in the authors’ past studies (*n* = 452, *M* = 110.48, *SD* = 33.77; *t* (490) = − 1.82, *p* = .069). Also, our participants’ performance on path integration, distance and direction deviations in pathway completion (around 100 cm and 15°, respectively), are smaller than the values observed in past studies (e.g., 168 cm and 24° reported by Loomis et al., 1993). That is, people with a poor sense of direction are not low on these abilities. These basic spatial aptitudes are probably necessary, but not sufficient, for successful cognitive mapping in large-scale environments. That is, large-scale spatial cognition deserves and requires investigation in its own right (Jacobs & Menzel, 2014; Wolbers & Wiener, 2014) beyond psychometric spatial ability and motion-based spatial updating (dead reckoning).

Our findings about the effects of training in spatial orientation suggest a possible approach to instruction in cognitive mapping. First, one can assure people with a poor sense of direction that they are not inferior in the basic spatial skills of either mental rotation or path integration. Second, it is informative and instructive to them if one explains that accurate spatial orientation involves an understanding of distance and direction and that the latter is often found difficult when flexible judgments of relative directions are required. Spatial orientation in the environment builds upon, but goes beyond, mental image rotation and spatial updating with respect to the immediate surround. Based on this premise, one can encourage people to practice distance and direction judgments with feedback. One should, at the same time, keep in mind that although practice in allocentric spatial orientation and path integration enhances people’s sensitivity to direction information, the effect is limited. This signifies the unique characteristic of cognitive mapping in large-scale environments and differences from instruction in landmark identification and route learning (e.g., Cornell, Heth, & Rowat, 1992; Nothegger, Winter, & Raubal, 2004).

The present results point to the distinction between distance and direction knowledge in the environment and revealed that the latter is specifically *orientational* in nature. Importantly, facility with directional judgments is resistant to an instructional treatment. To the extent that adaptive and successful behavior in the environment requires both egocentric (viewer-centered) and allocentric (environment-centered) representations (Burgess, 2006; Gallistel, 1990) and the flexible translation between them is difficult and not readily trainable, the issue of individual differences bears significance in the research into spatial cognition and should stimulate further studies from the viewpoint of skill training.

## Figures and Tables

**Fig. 7:**
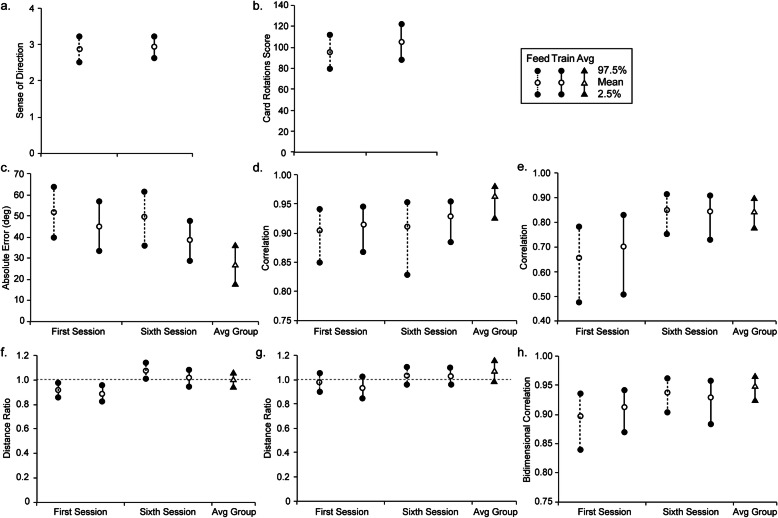
Results of a Bayesian analysis of participants’ attributes and performance: 95% credible intervals (circles represent participants with a poor sense of direction, dotted lines for the feedback-only condition and solid lines for the training condition, and triangles represent participants in the average group) for sense of direction (**a**), Card Rotations scores (**b**), direction estimates (**c**), distance correlations for route distances (**d**) and straight-line distances (**e**), distance ratios for route distances (**f**) and straight-line distances (**g**), and sketch maps (**h**)

**Fig. 8:**
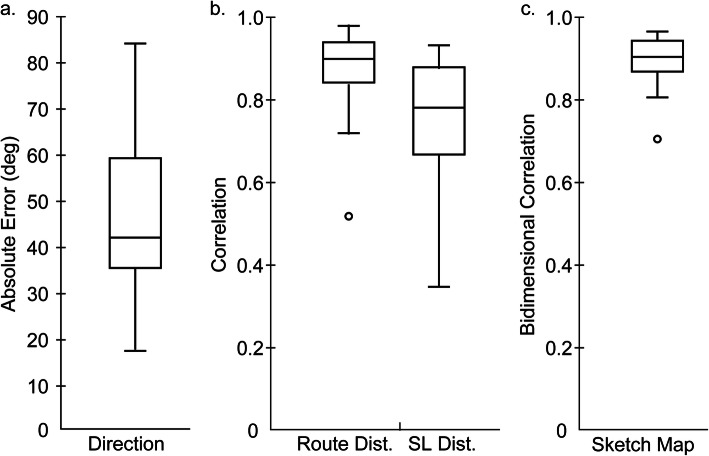
Boxplots of direction estimates (**a**), distance estimates (**b**), and sketch maps (**c**), for the participants with a poor sense of direction. Each box extends from the first to the third quartiles, with an internal horizontal line across the box indicating the median. The whiskers extend to the highest and lowest values within 1.5 times the interquartile range. Values outside the range are depicted by circles. SL Dist., straight-line distance

## Data Availability

The datasets used and/or analyzed during the current study are available from the corresponding author on reasonable request.

## Appendix A

### Bayesian analysis of participants’ attributes and performances

We further examined (a) the comparability of participants in the two experimental conditions and (b) the performances by participants with a poor and an average sense of direction in a Bayesian approach with Markov chain Monte Carlo methods. This was to corroborate the findings from the analyses above and deal with a caveat about the interpretation of null results in hypothesis testing in general.^6^ The results are shown in Fig. 7 (95% credible intervals for Bayesian estimates), which support the interpretations of the results in the preceding sections. Specifically, the posterior distributions of SBSOD and Card Rotations scores for the feedback-only and training conditions overlap for the most part (Fig. 7a and b). Participants in the training condition estimated directions more accurately than those in the feedback-only condition, but they did not perform as well as participants in the average group (Fig. 7c). Participants in the feedback-only and training conditions did not differ in the accuracy of route and straight-line distance estimates and sketch maps; by the sixth session, the accuracy of their distance estimates (straight-line distance correlations and route and straight-line distance ratios) and sketch maps became comparable to the accuracy for the average group (Figs. 7d–h).

## Appendix B

### Individual analysis of performances on spatial tasks

We also looked at the accuracy and development of individual participants’ direction estimates (absolute errors), distance estimates (distance correlations), and sketch maps (bidimensional correlation coefficients) over the six sessions. As shown in the box plots in Fig. 8, there are still fairly large individual differences among people with a poor sense of direction.

#### Direction estimates

The accuracy of direction estimates amomg 39 participants did not show a significant improvement over the sessions. Mean absolute errors for these participants ranged from 17.67° to 84.33°, with a mean of 46.2° and a standard deviation of 16.64°. One participant (female, in the feedback-only condition) showed an improvement: the developmental curve for this participant had a significant cubic trend, with an error decreasing from 83° in the first session to 21° in the last session.

#### Distance estimates

**Route distances** None of the participants showed a significant improvement in the accuracy of route distance estimates over the sessions. Their mean route distance correlations ranged from .52 to .98, with a mean of .88 and a standard deviation of .09.

**Straight-line distances** The accuracy of straight-line distance estimates among 36 participants did not show a significant improvement over the sessions. Mean straight-line distance correlations for these participants ranged from .35 to .93, with a mean of .75 and a standard deviation of .15. Four participants showed an improvement: the developmental curves for two participants (both female, in the feedback-only condition) had a significant linear trend, with a correlation increasing from .69 to .93 and from .02 to .96., and those for two participants (both male, in the training condition) had a significant cubic trend, with a correlation increasing from .08 to .98 and from .52 to .92.

#### Sketch maps

The accuracy of sketch maps for 38 participants did not show a significant improvement over the sessions. Mean bidimensional correlations for these participants ranged from .70 to .96, with a mean of .90 and a standard deviation of .06. Two participants (both male, in the feedback-only condition) showed an improvement: the developmental curves for these participants had a significant linear trend, with a bidimensional correlation increasing from .40 to .94 and from .16 to .95.

## Abbreviations

ANOVA: Analysis of variance
*M*: Mean
*SD*: Standard deviation
SOD: Sense of direction
SBSOD scale: Santa Barbara Sense-of-Direction scale
STEM: Science, technology, engineering, and mathematics

## Footnotes

1 In a power analysis for a between-subject effect in a mixed analysis of variance (two conditions), with an alpha level of 0.05, power of 0.80, and an effect size of 0.45 (the mean effect size, Cohen’s *f*, calculated from the present results), the necessary sample size was estimated to be 40.77.

2 In the Ishikawa and Montello (2006) study, configurational learning was not improved by mere repetition, and in general, training without feedback is not considered a desirable form of instructional intervention. Hence in the present experiment, feedback was given to participants in the training condition on how well they performed on the training tasks.

3 Another possible design would have given feedback on the experimental tasks only to participants in the feedback-only condition; namely, participants in the training condition would have received feedback only on the training tasks. In that design, however, a comparison of performances between the feedback-only and training conditions would not disentangle the effects of feedback on the training tasks and feedback on the experimental tasks. Thus, in the present experiment, feedback on the experimental tasks was given to participants in both conditions.

4 The pooled standard deviation was used as a standardizer. For paired *t* tests, the standardized mean difference effect size for within-subject designs, *d*_*z*_, was used (Lakens, 2013). As a rule of thumb, an effect-size value of 0.20 is interpreted as small, 0.50 as medium, 0.80 as large, and 1.30 as very large (Cohen, 1988).

5 The accuracy was high both in men and women, with a mean route distance correlation of .88 and .93, respectively, and so this difference is not discussed further.
